# Association of Histologic Disease Activity With Progression of Nonalcoholic Fatty Liver Disease

**DOI:** 10.1001/jamanetworkopen.2019.12565

**Published:** 2019-10-04

**Authors:** David E. Kleiner, Elizabeth M. Brunt, Laura A. Wilson, Cynthia Behling, Cynthia Guy, Melissa Contos, Oscar Cummings, Matthew Yeh, Ryan Gill, Naga Chalasani, Brent A. Neuschwander-Tetri, Anna Mae Diehl, Srinivasan Dasarathy, Norah Terrault, Kris Kowdley, Rohit Loomba, Patricia Belt, James Tonascia, Joel E. Lavine, Arun J. Sanyal

**Affiliations:** 1 Laboratory of Pathology, National Cancer Institute, National Institutes of Health, Bethesda, Maryland; 2 Department of Pathology and Immunology, Washington University School of Medicine in St Louis, St Louis, Missouri; 3 Department of Epidemiology, Bloomberg School of Public Health, Johns Hopkins University, Baltimore, Maryland; 4 Department of Pathology, University of California San Diego School of Medicine, San Diego; 5 Department of Pathology, Duke University, Durham, North Carolina; 6 Department of Pathology, Virginia Commonwealth University School of Medicine, Richmond; 7 Department of Pathology, Indiana University School of Medicine, Indianapolis; 8 Department of Pathology, University of Washington, Seattle; 9 Department of Pathology, University of California San Francisco School of Medicine, San Francisco; 10 Division of Gastroenterology and Hepatology, Indiana University School of Medicine, Indianapolis; 11 Division of Gastroenterology, Saint Louis University School of Medicine, St Louis, Missouri; 12 Department of Gastroenterology, Duke University, Durham, North Carolina; 13 Department of Gastroenterology and Hepatology, Cleveland Clinic, Cleveland, Ohio; 14 Division of Gastroenterology and Liver, University of Southern California, Los Angeles; 15 Liver Care Network and Organ Care Research, Swedish Medical Center, Seattle, Washington; 16 Division of Gastroenterology, University of California San Diego School of Medicine, San Diego; 17 Department of Biostatistics, Bloomberg School of Public Health, Johns Hopkins University, Baltimore, Maryland; 18 Division of Pediatric Gastroenterology, Department of Pediatrics, Columbia University, New York, New York; 19 Division of Gastroenterology and Hepatology, Virginia Commonwealth University School of Medicine, Richmond

## Abstract

**Importance:**

The histologic evolution of the full spectrum of nonalcoholic fatty liver disease (NAFLD) and factors associated with progression or regression remain to be definitively established.

**Objective:**

To evaluate the histologic evolution of NAFLD and the factors associated with changes in disease severity over time.

**Design, Setting, and Participants:**

A prospective cohort substudy from the Nonalcoholic Steatohepatitis Clinical Research Network (NASH CRN) NAFLD Database study, a noninterventional registry, was performed at 8 university medical research centers. Masked assessment of liver histologic specimens was performed, using a prespecified protocol to score individual biopsies. Participants included 446 adults with NAFLD enrolled in the NASH CRN Database studies between October 27, 2004, and September 13, 2013, who underwent 2 liver biopsies 1 or more year apart. Data analysis was performed from October 2016 to October 2018.

**Main Outcomes and Measures:**

Progression and regression of fibrosis stage, using clinical, laboratory, and histologic findings, including the NAFLD activity score (NAS) (sum of scores for steatosis, lobular inflammation, and ballooning; range, 0-8, with 8 indicating more severe disease).

**Results:**

A total of 446 adults (mean [SD] age, 47 [11] years; 294 [65.9%] women) with NAFLD (NAFL, 86 [19.3%]), borderline NASH (84 [18.8%]), and definite NASH (276 [61.9%]) were studied. Over a mean (SD) interval of 4.9 (2.8) years between biopsies, NAFL resolved in 11 patients (12.8%) and progressed to steatohepatitis in 36 patients (41.9%). Steatohepatitis resolved in 24 (28.6%) of the patients with borderline NASH and 61 (22.1%) of those with definite NASH. Fibrosis progression or regression by at least 1 stage occurred in 132 (30%) and 151 [34%] participants, respectively. Metabolic syndrome (20 [95%] vs 108 [72%]; *P* = .03), baseline NAS (mean [SD], 5.0 [1.4] vs 4.3 [1.6]; *P* = .005), and smaller reduction in NAS (−0.2 [2] vs −0.9 [2]; *P* < .001) were associated with progression to advanced (stage 3-4) fibrosis vs those without progression to stage 3 to 4 fibrosis. Fibrosis regression was associated with lower baseline insulin level (20 vs 33 μU/mL; *P* = .02) and decrease in all NAS components (steatosis grade −0.8 [0.1] vs −0.3 [0.9]; *P* < .001; lobular inflammation −0.5 [0.8] vs −0.2 [0.9]; *P* < .001; ballooning −0.7 [1.1] vs −0.1 [0.9]; *P* < .001). Only baseline aspartate aminotransferase (AST) levels were associated with fibrosis regression vs no change and progression vs no change on multivariable regression: baseline AST (regression: conditional odds ratio [cOR], 0.6 per 10 U/L AST; 95% CI, 0.4-0.7; *P* < .001; progression: cOR, 1.3; 95% CI, 1.1-1.5; *P* = .002). Changes in the AST level, alanine aminotransferase (ALT) level, and NAS were also associated with fibrosis regression and progression (ΔAST level: regression, cOR, 0.9; 95% CI, 0.6-1.2; *P* = .47; progression, cOR, 1.3; 95% CI, 1.0-1.6; *P* = .02; ΔALT level: regression, cOR, 0.7 per 10 U/L AST; 95% CI, 0.5-0.9; *P* = .002; progression, cOR, 1.0 per 10 U/L AST; 95% CI, 0.9-1.2; *P* = .93; ΔNAS: regression, cOR, 0.7; 95% CI, 0.6-0.9; *P* = .001; progression, cOR, 1.3; 95% CI, 1.1-1.5; *P* = .01).

**Conclusions and Relevance:**

Improvement or worsening of disease activity may be associated with fibrosis regression or progression, respectively, in NAFLD.

## Key Points

**Question:**

In patients with nonalcoholic fatty liver disease who are not undergoing specific therapeutic interventions, what factors are associated with progression and regression of hepatic fibrosis?

**Findings:**

In this cohort study of 446 patients, high baseline nonalcoholic fatty liver disease activity score and changes in the score were associated with concordant changes in fibrosis. Weight gain, high baseline aspartate aminotransferase level, and increases in the aspartate aminotransferase level were associated with fibrosis progression.

**Meaning:**

These data may support the use of therapeutics targeting disease activity in nonalcoholic steatohepatitis and the use of short-term changes in the nonalcoholic fatty liver disease activity score as an end point in such clinical trials; in clinical practice, weight gain and an increasing aspartate aminotransferase level should increase suspicion of increasing fibrosis.

## Teaser

This cohort study evaluates the progression and regression of hepatic fibrosis over time in association with the severity of nonalcoholic fatty liver disease.

## Introduction

Nonalcoholic fatty liver disease (NAFLD) is a leading cause of liver-related morbidity and mortality. Nonalcoholic steatohepatitis (NASH) is an aggressive form of NAFLD and is more likely to progress to cirrhosis compared with nonalcoholic fatty liver (NAFL).^1^ The growing contribution of NAFLD to the liver-related population burden of disease underscores the need for a granular understanding of the evolution of the disease and the factors associated with both disease progression and regression.

Nonalcoholic fatty liver disease can progress to NASH, and NASH can both progress and regress without specific pharmacologic intervention.^2,3,4,5,6^ However, there is uncertainty around the rates of change and the associated factors, especially when accounting for baseline phenotype (ie, NAFL vs steatohepatitis, disease activity, and stage). Prior studies are mostly single-center, retrospective experiences and limited by small sample sizes, which do not capture the full spectrum of disease, and variable or unreported case definitions.^7,8,9^ This lack of data has hindered development of generalizable models of disease evolution that are needed both to guide patient care and to conduct clinical trials designed to evaluate the effect of therapy on progression to cirrhosis, which is the generally accepted surrogate end point for drug approval.^10^ Lack of data further limits development of robust population burden of disease models needed to inform policies regarding resource allocation to treat this disease. There is therefore an unmet need for data derived from prospective, multicenter, protocol-driven assessment of the histologic evolution of NAFLD using validated scoring systems implemented in a standardized manner.

In this study, the histologic evolution of NAFLD and the factors associated with changes in disease severity over time were determined by analysis of prospectively collected clinical, laboratory, and histologic data from a cohort of participants with the full histologic spectrum of disease who underwent at least 2 liver biopsies more than 1 year apart.

## Methods

The NASH Clinical Research Network (CRN) is an ongoing National Institutes of Health–funded research network focused on better characterization of the clinical features and pathogenesis of NASH with the ultimate aim of developing improved means of its prevention and treatment. This analysis is a substudy of the NAFLD Database study (phases 1 and 2), a noninterventional registry of the network. The institutional review boards at each clinical center and the data coordinating center approved both protocols. All participants provided written informed consent. This study followed the Strengthening the Reporting of Observational Studies in Epidemiology (STROBE) reporting guideline for cohort studies.

### Patient Populations

Adults enrolled in the NAFLD Database study (phases 1 and 2) between October 27, 2004, and September 1, 2013, were included in this analysis if they had 2 evaluable biopsies 1 year or more apart. Histologic examination of liver biopsy specimens confirmed NAFLD in all cases. A daily consumption of alcohol less than 20 g for women and 30 g for men, as determined by the Alcohol Use Disorders Identification Test questionnaire,^11^ established the nonalcoholic nature of the liver disease. The baseline biopsy was either a historical biopsy performed prior to entry into the database or a liver biopsy performed within 90 days of enrollment. The patients received standard of care based on a networkwide standard-of-care document. Investigators performed follow-up biopsies based on clinical judgment. Individuals recruited into clinical trials from the NAFLD Database study and who were in the placebo arm of completed trials received a protocol-mandated biopsy and were included, while those in the active arm of such trials were excluded. Patients with nonevaluable biopsy samples (mean biopsy length, 5.2 mm) were excluded, as were those who underwent bariatric surgery or liver transplantation.

### Histologic Assessment

Hematoxylin-eosin–stained slides as well as Masson trichrome–stained slides were coded and read in a masked manner by the pathology committee of the NASH CRN as described previously.^12,13^ The liver histologic characteristics were assessed and the individual features of NAFLD were identified and scored as previously described; the interrater concordance statistics for the assessment of NASH have also been previously published.^12,14^ We have recently reassessed these as part of an internal quality control process for the CRN pathology committee. The κ statistics for the specific findings were as follows: steatosis, 0.77 (95% CI, 0.69-0.84); ballooning, 0.54 (95% CI, 0.44-0.65); lobular inflammation, 0.46 (95% CI, 0.34-0.58); NAFLD activity score (NAS), 0.52 (95% CI, 0.44-0.6); fibrosis, 0.75 (95% CI, 0.67-0.82); and steatohepatitis diagnosis, 0.66 (95% CI, 0.57-0.75).

Fatty liver (NAFL) was defined by steatosis with or without inflammation but without ballooning injury. This category also included some cases of steatosis with fibrosis without significant inflammation or ballooning in alignment with recent literature.^15^ Cases designated as borderline steatohepatitis had some but not all features of definite steatohepatitis, usually consisting of steatosis, inflammation, and appropriate fibrosis without ballooning. Definite steatohepatitis was defined by the presence of macrovesicular steatosis, inflammation, and hepatocellular ballooning in a mainly zone 3 distribution.^16^ Disease activity was determined from the severity of steatosis, lobular and portal inflammation, and characteristic hepatocellular ballooning. The NAS was computed by adding the severity scores for steatosis, lobular inflammation, and ballooning (range 0-8, with 8 indicating more severe disease).^12^

The minimum criteria for defining resolution of NASH was a disappearance of ballooning with modest or no residual inflammation.^15^ Resolution of NASH included those with resolution of NAFLD or residual NAFL. The definition of resolution of NAFLD included disappearance of hepatocellular ballooning with minimal or no lobular inflammation and a decrease in hepatic steatosis to less than 5% in the second biopsy.

### Clinical and Laboratory Data

All clinical and laboratory data were collected prospectively after the patient consented to be screened for enrollment in the NASH CRN. Because some participants’ biopsies were historical, analyses of the associations between histologic and clinical and laboratory data were limited to a subset of participants with clinical and laboratory data collected within 6 months of the biopsy.

### Statistical Analysis

Descriptive statistics were used to characterize individual groups of participants. Additional details are provided in the eMethods in the Supplement. Comparison of groups with or without steatohepatitis resolution or with fibrosis progression or regression was performed using *t* tests or analysis of variance for continuous measures, and Fisher exact test for categorical measures. Analysis of covariance adjusting for baseline values was used to compare changes in characteristics from the first to the second biopsy across groups. Nominal (ie, no adjustments for multiple comparisons) 2-sided *P* values were considered significant if *P* < .05.

A multinomial logistic regression model identified factors associated with fibrosis regression and progression. Fibrosis regression (decrease of ≥1 fibrosis stage, with change from stage 1b to 1a also considered regression), fibrosis progression (increase of ≥1 fibrosis stage, with change from stage 1a to 1b also considered progression), or no change (reference category) was the outcome variable. Conditional odds ratios (cORs), 95% CIs, and *P* values were calculated. The best subset of variables independently and parsimoniously associated with the outcome was determined as the subset of all possible models with the lowest Akaike information criteria.^17,18^

Three candidate variable sets associated with fibrosis change were included. The first set comprised demographic, clinical, and histologic model candidates (32 factors, 2^32^ models); the characteristics included age at biopsy (years), sex, race (white vs nonwhite), Hispanic/Latino ethnicity, diabetes status, metabolic syndrome, current smoker, and use of metformin, vitamin E, and statins. This model also included baseline values and change from baseline in the following variables: body mass index (BMI) (calculated as weight in kilograms divided by height in meters squared); alanine aminotransferase (ALT) (units per liter), aspartate aminotransferase (AST) (units per liter), alkaline phosphatase (units per liter), low-density lipoprotein cholesterol (milligrams per deciliter), high-density lipoprotein cholesterol (milligrams per deciliter), triglyceride levels (milligrams per deciliter), and homeostatic model assessment of insulin resistance (glucose [millimoles per liter] × insulin [micro units per milliliter divided by 22.5]). The histologic variables in this model included baseline steatosis grade, lobular inflammation grade, ballooning grade, presence of Mallory-Denk bodies, portal inflammation grade, and fibrosis stage.

The second model included only histologic variables (11 factors, 2^11^ models). The model included baseline fibrosis stage and baseline values and change in steatosis grade, lobular inflammation grade, ballooning grade, presence of Mallory-Denk bodies, and portal inflammation grade. A third model also evaluated histologic variables only and differed from the second model by including the NAS while excluding its individual components (n = 7, 2^7^ models). Time between biopsies (years) was included in all analyses. Analyses were performed using SAS, version 9.4, software (SAS Institute Inc) and Stata, release 15.1 (StataCorp). Data analysis was performed from October 2016 to October 2018.

## Results

**Figure 1.**
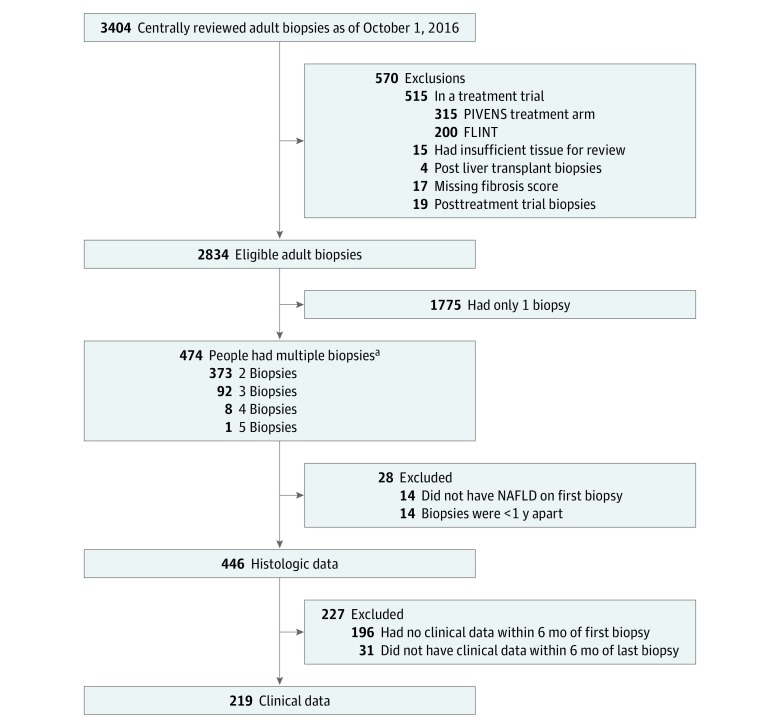
Flow Diagram Showing Participants Evaluated and Included in the Study FLINT indicates the Farnesoid X Receptor Ligand Obeticholic Acid in NASH Treatment Trial; PIVENS, Pioglitazone versus Vitamin E versus Placebo for the Treatment of Nondiabetic Patients with Nonalcoholic Steatohepatitis Trial; and NAFLD, nonalcoholic fatty liver disease. ^a^The total number of biopsies was 1059.

A total of 3404 evaluable liver biopsy results were available (Figure 1). The principal exclusions were the presence of only a single biopsy for a given individual (n = 1775) or participation in the active arm of a clinical trial (n = 515). A total of 446 adult patients met entry criteria, with mean (SD) age at first biopsy 47 (11) years; of these, 294 (65.9%) were women, 374 of 425 individuals (88.0%) reporting race were white, 40 (9.0%) were of Hispanic/Latino ethnicity, and mean (SD) BMI was 35 (7). Individuals whose biopsies demonstrated definite NASH, compared with those with NAFL, were older (mean [SD], 49 [11] vs 45 [11] years; *P* = .007), were more likely to have diabetes (115 of 276 [41.7%] vs 18 of 86 [20.9%]; *P* < .001) and metabolic syndrome (109 of 142 [76.8%] vs 21 of 38 [55.3%]; *P* = .03), and had higher liver enzyme levels (eg, mean [SD] ALT, 87 [61] vs 53 [29] U/L [to convert to microkatals per liter, multiply by 0.0167]; *P* = .002) (Table 1). The interval between biopsies was longer in those with NAFL vs those with definite NASH (mean [SD], 5.9 [2.8] vs 4.6 [2.7] years; *P* = .001) on the baseline biopsy. A subset of patients (n = 219) had clinical and laboratory data obtained within 6 months of both index and follow-up biopsies and were used to model the factors associated with change in histologic severity from the first to last biopsy. This subset was comparable to the overall cohort (eTable 1 in the Supplement) with the exception of a shorter interval (mean [SD], 3.9 [1.2] vs 4.9 [2.8] years) between biopsies.

**Table 1.** Baseline Characteristics by Steatohepatitis Diagnosis at Screening^a^

Patient Characteristics	NAFL	Steatohepatitis		*P* Value^b^
		Borderline	Definite	
**Demographics**				
No.	86	84	276	
Age at biopsy, mean (SD), y	45 (11)	45 (11)	49 (11)	.007
Male, No. (%)	34 (39.5)	34 (40.5)	84 (30.4)	.12
White race, No. (%)^c^	74 (89.2)	71 (89.9)	229 (87.1)	.81
Hispanic or Latino, No. (%)	10 (11.6)	6 (7.1)	24 (8.7)	.60
Diabetes, No. (%)	18 (20.9)	16 (19.0)	115 (41.7)	<.001
Smoking history, No. (%)				
Current smoker	9 (10.5)	13 (15.5)	18 (6.5)	.04
Ever smoked	28 (32.6)	34 (40.5)	101 (36.6)	.57
**Clinical and Laboratory Data**^d^				
No.	38	39	142	
Metabolic syndrome, No. (%)	21 (55.3)	29 (76.3)	109 (77.3)	.03
BMI, mean (SD)	34.1 (7.1)	34.5 (5.3)	35.9 (6.9)	.24
ALT, mean (SD), U/L	53 (29)	73 (39)	87 (61)	.002
AST, mean (SD), U/L	38 (22)	45 (25)	65 (51)	<.001
Alkaline phosphatase, mean (SD), U/L	84 (23)	76 (22)	88 (24)	.02
Cholesterol, mean (SD), mg/dL				
LDL^c^	125 (37)	121 (42)	123 (34)	.90
HDL	48 (11)	41 (10)	42 (10)	.004
Triglycerides	161 (78)	152 (65)	194 (121)	.04
Glucose, mean (SD), mg/dL	95 (18)	102 (24)	105 (35)	.17
Insulin, mean (SD), μU/mL^c^	17 (10)	23 (27)	28 (33)	.13
HOMA-IR, mean (SD)^e^	4.3 (3.0)	5.8 (6.5)	7.9 (11.3)	.08
Medication use, No. (%)				
Metformin	3 (7.9)	9 (23.1)	34 (23.9)	.08
Vitamin E	4 (10.5)	7 (17.9)	22 (15.5)	.68
Statins	7 (18.4)	10 (25.6)	33 (23.2)	.75
**Histologic Findings**				
No.	86	84	276	
Hepatic steatosis (numeric score), No. (%)				
0 (<5%)	0	3 (3.6)	2 (0.7)	<.001
1 (5%-33%)	52 (60.5)	28 (33.3)	87 (31.5)	
2 (34%-66%)	22 (25.6)	21 (25.0)	104 (37.7)	
3 (>66%)	12 (14.0)	32 (38.1)	83 (30.1)	
Steatosis score, mean (SD)^f^	1.5 (0.7)	2.0 (0.9)	2.0 (0.8)	<.001
Lobular inflammation score , foci per 20× high power field, No. (%)				
0 (None)	1 (1.2)	0	0	<.001
1 (<2)	70 (81.4)	48 (57.1)	96 (34.8)	
2 (2-4)	13 (15.1)	31 (36.9)	133 (48.2)	
3 (>4)	2 (2.3)	5 (6.0)	47 (17.0)	
Lobular inflammation score, mean (SD)^g^	1.2 (0.5)	1.5 (0.6)	1.8 (0.7)	<.001
Ballooning, No. (%)				
0 (None)	84 (97.7)	47 (56.0)	0	<.001
1 (Few)	2 (2.3)	34 (40.5)	86 (31.2)	
2 (Many)	0	3 (3.6)	190 (68.8)	
Ballooning score, mean (SD)^h^	0.0 (0.2)	0.5 (0.6)	1.7 (0.5)	<.001
Mallory-Denk bodies, No. (%)	0	3 (3.6)	138 (50)	<.001
Portal inflammation score, No. (%)				
0 (None)	31 (36.0)	13 (15.5)	24 (8.7)	<.001
1 (Mild)	47 (54.7)	57 (67.9)	173 (62.7)	
2 (More than mild)	8 (9.3)	14 (16.7)	79 (28.6)	
Portal inflammation score, mean (SD)^i^	0.7 (0.6)	1.0 (0.6)	1.2 (0.6)	<.001
Fibrosis stage, No. (%)				
None	66 (76.7)	27 (32.1)	15 (5.4)	<.001
1a	7 (8.1)	19 (22.6)	31 (11.2)	
1b	2 (2.3)	5 (6.0)	49 (17.8)	
1c	7 (8.1)	7 (8.3)	1 (0.4)	
2	2 (2.3)	12 (14.3)	72 (26.1)	
3	2 (2.3)	12 (14.3)	92 (33.3)	
4	0	2 (2.4)	16 (5.8)	
Fibrosis stage, mean (SD)	0.3 (0.6)	1.2 (1.1)	2.0 (1.0)	<.001
NAS, mean (SD)^j^	2.7 (0.9)	3.9 (1.2)	5.5 (1.2)	<.001
Interval between biopsies, mean (SD), y	5.9 (2.8)	5.0 (2.9)	4.6 (2.7)	.001
Biopsy length, mean (SD), mm	17.6 (8.3)	17.6 (8.1)	19.2 (9.8)	.21

Abbreviations: ALT, alanine aminotransferase; AST, aspartate aminotransferase; BMI, body mass index (calculated as weight in kilograms divided by height in meters squared); HDL, high-density lipoprotein; HOMA-IR, homeostatic model assessment of insulin resistance; LDL, low-density lipoprotein; NAFL, nonalcoholic fatty liver; NAS, nonalcoholic fatty liver disease activity score.

SI conversion factors: To convert alkaline phosphatase, ALT, and AST to microkatals per liter, multiply by 0.0167; glucose to millimoles per liter, multiply by 0.0555; HDL and LDL to millimoles per liter, multiply by 0.0259; insulin to picomoles per liter, multiply by 6.945; and triglycerides to millimoles per liter, multiply by 0.0113.

^a^ The numbers of patients for clinical and laboratory data are smaller because only data collected within 6 months of the biopsy were included (n = 446 with demographic and histologic data; n = 219 with clinical data collected within 6 months of biopsy).

^b^ *P* values derived from Fisher exact test for categorical variables and analysis of variance for continuous measures.

^c^ Twenty-one participants refused to provide their race, 4 participants were missing LDL cholesterol level, and 3 participants were missing insulin and HOMA-IR values.

^d^ Data collected within 6 months of biopsy. In the Metabolic syndrome for Borderline category, 2 patients were missing metabolic syndrome (1 in the borderline category and 1 in the definite category).

^e^ Determined as glucose (millimoles per liter) × insulin (micro units per mililiter divided by 22.5).

^f^ Steatosis score ranges from 0 (<5% hepatic steatosis) to 3 (>66% hepatic steatosis).

^g^ Lobular inflammation score ranges from 0 (no foci per ×20 high power) to 3 (>4 foci per ×20 high power).

^h^ Ballooning score ranges from 0 (no ballooned cells) to 2 (many ballooned cells).

^i^ Portal inflammation score ranges from 0 (no portal inflammation) to 2 (more than mild portal inflammation).

^j^ Sum of scores for steatosis, lobular inflammation, and ballooning; range, 0 to 8, with 8 indicating more severe disease.

### Bidirectional Evolution of NAFL and NASH

**Figure 2.**
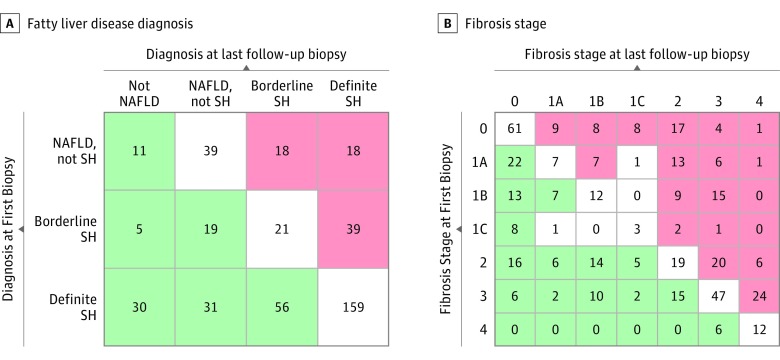
Examples of Progression and Regression of Nonalcoholic Fatty Liver Disease (NAFLD) Changes in diagnosis and fibrosis from first biopsy to last showing numbers of patients with changes of diagnosis (A) or fibrosis stage (B). Green boxes indicate improved diagnosis or regression of fibrosis; red boxes indicate worsening diagnosis or progression of fibrosis. White boxes indicate either no change in diagnosis or fibrosis stage or, in the case of off-diagonal boxes (B), a change in fibrosis that cannot be clearly defined as progression or regression.^19^ SH indicates steatohepatitis.

Eleven of 86 patients (12.8%) with NAFL on the baseline biopsy had complete resolution of NAFLD, whereas 36 (41.9%) progressed to either borderline (18 [20.9%]) or definite (18 [20.9%]) steatohepatitis (Figure 2A). A greater proportion of 84 patients with borderline steatohepatitis on the first biopsy progressed to definite steatohepatitis (39 [46.4%]) than regressed to NAFL (84 [22.6%]) or resolved NAFLD (5 [6.0%]). Thirty of 276 participants’ biopsies (10.9%) with definite steatohepatitis had complete resolution of NAFLD, whereas 56 (20.3%) regressed to borderline steatohepatitis and 31 (11.2%) regressed to NAFL.

A total of 85 of 360 participants (23.6%) whose biopsy results showed borderline or definite steatohepatitis had resolution of NASH over the observation time (mean [SD], 5.0 [2.9] vs 4.6 [2.7] years for borderline and definite NASH, respectively). Those with resolved steatohepatitis had lower baseline mean (SD) AST (48 [31] vs 64 [50] U/L [to convert to microkatals per liter, multiply by 0.0167]; *P* = .01), lobular inflammation (1.6 [0.7] vs 1.8 [0.7]; *P* = .003) and fibrosis (1.4 [1.1] vs 2.0 [1.1]; *P* < .001) scores, lower presence of Mallory-Denk bodies (22 [26%] vs 119 [43%]; *P* = .005), as well as lower NAS (4.8 [1.4] vs 5.2 [1.4]; *P* = .009) (eTable 2 in the Supplement). Several clinical and laboratory variables tracked NASH resolution on univariate analysis. Compared with patients without NASH resolution, those with NASH resolution had a greater decrease in body weight (4.4-kg loss vs 1.2-kg gain; *P* < .001) and BMI (decrease of 1.4 vs increase of 0.5; *P* < .001). Those with NASH resolution also experienced a greater decrease in AST (20- vs 14-U/L decrease; *P* < .001), ALT (33-U/L vs 26-U/L decrease; *P* < .001), and fasting insulin levels (4.1-μU/mL decrease vs 0.4-μU/mL increase [to convert to picomoles per liter, multiply by 6.945]; *P* = .05). The only clinical measures associated with complete resolution of NAFLD were a decrease in weight and BMI.

**Figure 3.**
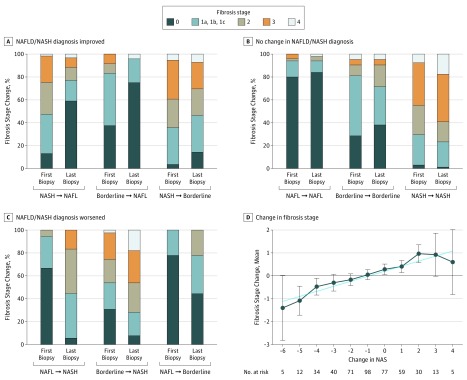
Histologic Evolution of Nonalcoholic Fatty Liver Disease (NAFLD) A-C, The proportion of patients with fibrosis stage 0, 1a, 1b, 1c, 2, 3, or 4 at first biopsy and last biopsy is shown in patients whose diagnosis improved, did not change, and worsened. A, For those with improvement in diagnosis, fibrosis stage is shown for those with diagnosis change from nonalcoholic steatohepatitis (NASH) to nonalcoholic fatty liver (NAFL) (n = 61), borderline to NAFL (n = 24), and NASH to borderline (n = 56). B, For patients with no change in diagnosis, fibrosis stage is shown for NAFL (n = 50) borderline (n = 21), and NASH (n = 159). C, For patients with worsening of diagnosis, fibrosis stage is shown for those with diagnosis change from NAFL to NASH (n = 18), borderline to NASH (n = 39), and NAFL to borderline (n = 18). D, The change in fibrosis stage from the first biopsy to the second is shown as a function of change in the NAFLD activity score (NAS). A reduction in the NAS was associated with a decrease in fibrosis stage, whereas an increase in the NAS was associated with an increase in fibrosis stage. Specifically, a 2-point or greater decrease in NAS was associated with fibrosis regression. One participant with a change in NAS of −7 and 1 participant with a change in NAS of +5 were removed from the figure. Spearman correlation coefficient, 0.38; slope, +0.67; SE, 0.08; *P* < .001. Error bars indicate 95% CIs.

Resolution of NASH, compared with nonresolved NASH, was also associated with a significant decrease in fibrosis stage (OR, 0.42; 95% CI, 0.32-0.54; *P* < .001) (Figure 3A). However, if the phenotype (NAFL or NASH) did not change, fibrosis stage did not change significantly (Figure 3B). Progression from NAFL to borderline NASH or from borderline to definite NASH was associated with fibrosis progression. Of those with NAFL progression, 36 of 57 patients (63.2%) had at least 1-stage fibrosis progression vs without NAFL progression and 108 of 389 (27.8%) had at least 1-stage fibrosis progression (*P* < .001) (Figure 3C).

### Disease Progression in Varying Phenotypes of NAFLD

Fibrosis increased by 1 or more stages in 151 (33.9%) of the entire cohort, in comparison with those with baseline stages of 0 (47 [44%]), 1 (47 [37%]), 2 (26 [30%]), and 3 (24 [23%]) (Figure 2B and eFigure in the Supplement). A greater proportion of participants with bridging fibrosis (stage 3) progressed to cirrhosis compared with those who had lower stages. Of those who had NAFL and stage 0 to 1 fibrosis at baseline, progression to stage 2 or greater fibrosis was associated with the appearance of steatohepatitis in the second biopsy (OR, 7.2; 95% CI, 3.1-25.9; *P* < .001). The factors associated with fibrosis change included white race (regression, 102 [82%]; no change, 138 [90%]; progression, 134 [91%]; *P* = .04) and current smoking (regression: 5 [4%], no change: 17 [10%], progression: 18 [12%]; *P* = .03) (eTable 3 in the Supplement). Those with fibrosis regression had a small but statistically significantly higher NAS at baseline (mean [SD] regression, 5.1 [1.5]; no change, 4.4 [1.7]; progression, 4.6 [1.5]; *P* = .002).

Fifty-four of 322 participants (16.8%) showed progression from stages 0 to 2 on the initial biopsy to advanced fibrosis (stages 3 and 4) on the follow-up liver biopsy. These individuals were more likely to have metabolic syndrome (20 [95%] vs 108 [72%]; *P* = .03) and higher mean (SD) baseline ALT (91 [45] vs 70 [46] U/L; *P* = .05) and AST (75 [57] vs 48 [39] U/L; *P* = .05) levels (eTable 4 in the Supplement). Participants with progression to advanced fibrosis also had higher baseline ballooning (mean [SD], 1.4 [0.8] vs 0.8 [0.8]; *P* < .001), presence of Mallory-Denk bodies (mean [SD], 24 [44%] vs 36 [13%]; *P* < .001), portal inflammation (mean [SD], 1.1 [0.5] vs 0.9 [0.6]; *P* = .001), fibrosis (mean [SD], 1.4 [0.7] vs 0.8 [0.8]; *P* < .001), and NAS (mean [SD], 5.0 [1.4] vs 4.3 [1.6]; *P* = .005) on the initial biopsy. Progression to advanced fibrosis was associated with either less improvement or worsening of AST levels (mean [SD], −6 [60] vs −11 [41] U/L change; *P* < .001), ALT levels (mean [SD], −14 [48] vs −20 [46] U/L change; *P* < .001), lobular inflammation (mean [SD], 0.0 [0.9] vs −0.3 [0.8] grade change; *P* < .001), ballooning (mean [SD], 0.3 [1.1] vs −0.2 [1.0] grade change; *P* < .001), Mallory-Denk bodies (mean [SD], 19 [35%] vs 21 [8%] worsened; *P* < .001), portal inflammation (mean [SD], 0.4 [0.8] vs 0.1 [0.7] grade change; *P* < .001), and overall NAS (mean [SD], −0.2 [2.0] vs −0.9 [2.0] point change; *P* < .001).

### Regression of Fibrosis Stage

The fibrosis stage decreased by 1 or more stages in 132 of 338 participants (39.1%) with at least stage 1 fibrosis at baseline (eFigure in the Supplement). Those with fibrosis regression had lower mean baseline fasting insulin levels (mean [SD], 20 [11] vs 33 [41] μU/mL; *P* = .02) and a greater decrease in AST and ALT levels from the time of the first to last biopsy (eTable 5 in the Supplement). A decrease in weight of 5 kg or more was associated with improvement of steatosis, lobular inflammation, and ballooning (eTable 6 in the Supplement). A decrease in all of the histologic markers of disease activity (steatosis, lobular and portal inflammation, ballooning, and Mallory-Denk bodies) and the composite NAS between the initial and final biopsies were all significantly associated with fibrosis regression (steatosis grade, −0.8 [0.1] vs −0.3 [0.9]; *P* < .001; lobular inflammation, −0.5 [0.8] vs −0.2 [0.9]; *P* < .001; portal inflammation, −0.1 [0.7] vs 0.2 [0.6]; *P* < .001; ballooning, −0.7 [1.1] vs −0.1 [0.9]; *P* < .001); improvement in presence of Mallory-Denk bodies, 31 [23%] vs 37 [18%]; *P* = .004; NAS, −2.0 [2.1] vs −0.6 [1.9]; *P* < .001).

### Factors in Fibrosis Progression and Regression

Of modeling factors associated with fibrosis change from index to last biopsy, the best subset of clinical and histologic factors included the following baseline variables: age at biopsy, serum AST level, current smoking, homeostatic model assessment of insulin resistance, portal inflammation and fibrosis stage, and change in ALT and AST levels (last biopsy vs first biopsy). The cOR of fibrosis regression vs no change decreased 40% per 10-U/L increment in baseline AST values (cOR, 0.6 per 10 U/L AST; 95% CI 0.4-0.7; *P* < .001). Conversely, the odds of fibrosis progression vs no change increased 30% per 10-U/L increment in baseline AST level (cOR, 1.3 per 10 U/L AST; 95% CI, 1.1-1.5; *P* = .002). Also, for every 10-U/L increase in AST level from the first to last biopsy, the odds of fibrosis progression by at least 1 stage increased by 30% (cOR, 1.3; 95% CI, 1.0-1.6; *P* = .02) but was not associated with fibrosis regression (cOR, 0.09; 95% CI, 0.6-1.2; P = 0.47). Increases in ALT level from first to last biopsy reduced the likelihood of fibrosis regression by 30% (cOR, 0.7; 95% CI, 0.5-0.9; *P* = .002), but was not significantly associated with fibrosis progression (cOR, 1.0; 95% CI, 0.9-1.2; *P* = .93). Active smokers also had a lower rate of fibrosis regression, compared with no change (cOR, 0.1; 95% CI, 0.0-0.5; *P* = .009).

Hepatocellular ballooning (cOR for fibrosis regression vs no change: 0.6; 95% CI, 0.3-0.9; *P* = .02; cOR for fibrosis progression vs no change: 3.7; 95% CI, 2.2-6.0; *P* < .001), portal inflammation (cOR for fibrosis regression vs no change: 0.4; 95% CI, 0.2-0.8; *P* = .007; cOR for fibrosis progression vs no change: 4.0; 95% CI, 2.1-7.7; *P* < .001), and fibrosis stage at baseline (cOR for fibrosis regression vs no change: 2.0; 95% CI, 1.5-2.8; *P* < .001; cOR for fibrosis progression vs no change: 0.4; 95% CI, 0.3-0.5; *P* < .001) were all associated with fibrosis regression or progression in models with histologic factors (Table 2). Increases in steatosis between biopsies decreased the odds of fibrosis regression but did not appear to be associated with progression. Increments and decrements in ballooning scores and prevalence of Mallory-Denk bodies from baseline to final biopsies were associated with fibrosis regression and progression respectively. Changes in portal inflammation from index to last biopsy also were associated with similar changes in fibrosis. Higher baseline NAS reflected increased risk of fibrosis progression (cOR, 1.3; 95% CI, 1.0-1.6; *P* = .04). Improvement in the NAS over time was associated with fibrosis regression (cOR, 0.7; 95% CI, 0.6-0.9; *P* < .001), and worsening of the NAS was associated with fibrosis progression (cOR, 1.3; 95% CI, 1.1-1.5; *P* < .001), compared with no change (Table 2, eFigure in the Supplement). Considering all substages of fibrosis stage 1 as a single stage did not significantly alter the results of the analysis.

**Table 2.** Multivariable Models for Clinical and Histologic Factors Associated With Fibrosis Regression and Progression

Variable	cOR (95% CI)^a^			
	Fibrosis Regression vs No Change	*P* Value	Fibrosis Progression vs No Change	*P* Value
**First Model: Clinical and Histologic (n = 216)**^b^				
Baseline clinical and histologic features				
Age at biopsy (per 10 y)	0.7 (0.5-1.0)	.05	1.3 (0.9-1.8)	.10
Current smoker	0.1 (0.0-0.5)	.009	2.4 (0.8-7.2)	.12
AST (per 10 U/L)	0.6 (0.4-0.7)	<.001	1.3 (1.1-1.5)	.002
HOMA-IR (per 5 U)	0.5 (0.4-0.8)	.003	1.1 (0.9-1.3)	.25
Portal inflammation	0.4 (0.2-0.9)	.02	1.4 (0.7-2.7)	.36
Fibrosis stage	3.0 (1.8-4.8)	<.001	0.6 (0.4-0.8)	.007
Change^c^				
ΔALT (per 10 U/L)	0.7 (0.5-0.9)	.002	1.0 (0.9-1.2)	.93
ΔAST (per 10 U/L)	0.9 (0.6-1.2)	.47	1.3 (1.0-1.6)	.02
Years between biopsies	1.1 (0.9-1.3)	.56	1.2 (1.0-1.4)	.04
**Second Model: Histologic (n = 445)**^d^				
Baseline				
Ballooning	0.6 (0.3-0.9)	.02	3.7 (2.2-6.0)	<.001
Portal inflammation	0.4 (0.2-0.8)	.007	4.0 (2.1-7.7)	<.001
Fibrosis stage	2.0 (1.5-2.8)	<.001	0.4 (0.3-0.5)	<.001
Change (last biopsy vs first)				
ΔSteatosis grade	0.6 (0.4-0.8)	<.001	0.8 (0.6-1.1)	.24
ΔBallooning	0.6 (0.4-0.8)	.002	3.0 (2.0-4.4)	<.001
ΔLobular inflammation	0.8 (0.6-1.2)	.33	1.3 (0.9-1.8)	.14
ΔPortal inflammation	0.5 (0.3-0.9)	002	4.1 (2.3-7.3)	<.001
Years between biopsies	0.9 (0.9-1.0)	.24	1.1 (1.0-1.2)	.07
**Third Model: Histologic (n = 445)**^e^				
Baseline				
NAS	1.1 (0.9-1.4)	.50	1.3 (1.0-1.6)	.04
Mallory-Denk bodies	0.2 (0.1-0.6)	.002	3.8 (1.5-9.7)	.005
Portal inflammation	0.4 (0.2-0.8)	.006	4.0 (2.1-7.7)	<.001
Fibrosis stage	2.0 (1.5-2.7)	<.001	0.4 (0.3-0.5)	<.001
Change (last biopsy vs first)				
ΔNAS	0.7 (0.6-0.9)	.001	1.3 (1.1-1.5)	.01
ΔMallory-Denk bodies	0.5 (0.1-1.1)	.07	2.5 (1.2-5.0)	.01
ΔPortal inflammation	0.5 (0.3-0.9)	.02	4.3 (2.5-7.5)	<.001
Years between biopsy	1.0 (0.9-1.1)	.47	1.1 (1.0-1.2)	.08

Abbreviations: ALT, alanine aminotransferase; AST, aspartate aminotransferase; cOR, conditional odds ratio; HOMA-IR, homeostatic model assessment of insulin resistance; NAS, nonalcoholic fatty liver disease activity score (sum of scores for steatosis, lobular inflammation, and ballooning; range, 0-8).

^a^ Determined with multivariable multinomial regression.

^b^ Factors associated with the first model are explained in the second and third paragraphs of the Statistical Analysis subsection of the Methods section. Total number is 216 owing to 3 participants missing HOMA-IR.

^c^ Change (value at last biopsy – first biopsy), where a positive value for change indicates worsening.

^d^ Factors associated with the second model are explained in the last paragraph of the Statistical Analysis subsection of the Methods section.

^e^ The third model differed from the second model as described in the last paragraph of the Statistical Analysis subsection of the Methods section.

## Discussion

A common belief is that NASH is a steadily progressive disorder resulting in cirrhosis. The present study appears to confirm and extend emerging data to shift this paradigm and reveal the dynamic nature of both disease activity and fibrosis stage and their close and concordant association.

Baseline steatosis, ballooning, and portal inflammation were all associated with both fibrosis progression and regression (Table 2). High NAS was associated with progression to advanced fibrosis (stages 3-4), and an improvement in the score was associated with a decrease in fibrosis stage. Spontaneous resolution of NASH or regression to NAFL was also associated with significant improvement in fibrosis. Conversely, development and progression of fibrosis in patients with an NAFL alone was associated with development of steatohepatitis. Together, these data suggest that not only is disease evolution dynamic, but the trajectory of fibrosis change is directly associated with changes in disease activity.

It is commonly believed that a minimum of 7% weight loss is needed for NASH resolution; however, the degree of mean weight loss in participants with NASH resolution was more modest in this study (−4.4 vs 1.2 kg from baseline to last biopsy for those with and without resolution). This relatively small change in weight is, however, not out of line with literature in which almost a third of patients with NASH resolution in a lifestyle intervention study had less than 7% weight loss.^20^ Weight change, categorized in terms of absolute changes of 5 kg from baseline, also tracked changes in the NAS in both directions (eTable 6 in the Supplement).

Resolution of NASH was also associated with weight loss (mean, −4.4 vs 1.2 kg from baseline to last biopsy for patients with and without resolution). However, there were no significant differences in weight between those with or without fibrosis regression (mean, −0.6 and 0.4 kg). These differences suggest that there are factors besides changes in weight that are associated with fibrosis regression in those with modest weight changes. Most participants with fibrosis regression, however, had less than 10% weight loss and this commonly believed relationship cannot be tested in this cohort.^20^

Most trials in patients with precirrhotic stages of NAFLD include those with high activity scores because a high level of disease activity is expected to reflect an increased risk of fibrosis progression. The baseline NAS in the present study was higher in patients with fibrosis regression than in those with progression. This apparent paradox most likely reflects the inclusion of patients with advanced fibrosis, which is associated with decreased disease activity^21^; when only those with stage 0 to 2 fibrosis at baseline were considered, a high NAS was associated with greater progression to advanced (stage 3-4) fibrosis.

Some studies have challenged the utility of assessment of histologic markers of activity in NASH.^3,22,23^ A priori, disease activity may be a factor in fibrosis stage, which reflects how far the disease has progressed to cirrhosis.^12^ It is therefore not surprising that studies focused on cirrhosis-related clinical outcomes have demonstrated a stronger association with fibrosis than markers of disease activity.^23^ The present study differs from these reports by its use of prespecified, protocol-driven prospective assessment of histologic changes and using changes in fibrosis rather than clinical outcomes as the measured readout of disease progression. The findings suggest a close association between disease activity and changes in disease activity to changes in fibrosis; this association appears to support the use of short-term improvement in disease activity as a surrogate end point estimation of reduced fibrosis progression in clinical trials of drugs that reduce disease activity. The findings further may support the validity of the use of the NAS for this purpose by linking changes in the NAS with changes in fibrosis stage.

Another problem in trial design is how to minimize placebo response. Patients with spontaneous resolution of NASH had lower baseline AST levels; conversely, higher AST levels were associated with a greater risk of progression to advanced fibrosis. Using an AST rather than an ALT threshold level for entry into trials may maximize differences between treatment and control arms.

This study also has potential implications for clinical practice. These data may provide support for current recommendations regarding weight loss as an important therapeutic objective to improve underlying NASH. In the presence of weight gain, particularly accompanied by increasing AST levels, the findings appear to support the need for added attention to the possibility of disease progression in clinical practice. While histologic assessment is the current reference standard for assessment of fibrosis progression, this standard will likely be replaced by noninvasive methods of disease assessment once they are qualified as measures of disease activity and stage. In addition, active smokers had a lower likelihood of fibrosis regression; this finding appears to corroborate the harmful effects of smoking.

###  Limitations

This study has limitations. A limitation of this study is that the indication for a follow-up biopsy was not standardized and protocol driven, thus introducing a potential bias toward overestimation of disease progression rates. The histologic transition rates from this convenience data set may thus not represent the rates in the general population. However, the performance of protocol biopsies also introduces potential bias owing to changes in patient behavior when they know a liver biopsy is imminent. This study also did not account for the potential contribution of low levels of alcohol use to the outcomes. Modest alcohol consumption lowers the likelihood of NASH resolution and is associated with more steatosis but without a demonstrable association with fibrosis.^24^ The predominantly white population in this study limits the generalizability of the results to other populations. Even with these limitations, these data suggest the dynamic nature of changes in disease activity and fibrosis over time in patients with NAFLD regardless of the initial histologic findings.

## Conclusions

The results of the present study suggest that the natural course of NAFLD is dynamic and characterized by waxing and waning of disease activity. These data further suggest an association between the direction and degree of change in histologic features of disease activity with fibrosis progression or regression independent of changes in body weight. These results may provide a rationale to focus future therapeutic development on modulation of disease activity and use the NAS as a surrogate end point in the short-term in clinical trials of NASH using agents that improve disease activity.

## Notes

Supplement. **eMethods.** Statistical Analysis **eTable 1.** Baseline Characteristics of Histology Group and Subset With Full Clinical Meta-Data **eTable 2.** Factors Associated With Resolution of NASH **eTable 3.** Baseline Characteristics by Fibrosis Evolution (Regressed, Stable, Progressed) **eTable 4.** Factors Associated With Progression to Advanced Fibrosis (Stage 3 or 4) in Patients With NAFLD on First Biopsy **eTable 5.** Factors Associated With Fibrosis Regression in Patients With Stage ≥1 Fibrosis and NAFLD on First Biopsy **eTable 6.** Histologic Features and Weight Change **eFigure.** Examples of Progression and Regression of Nonalcoholic Fatty Liver Disease
