# Supplementary material for: Association of Histologic Disease Activity With Progression of Nonalcoholic Fatty Liver Disease
Source: JAMA Netw Open. 2019 Oct 4;2(10):e1912565. doi: 10.1001/jamanetworkopen.2019.12565 (PMC6784786; doi:10.1001/jamanetworkopen.2019.12565)
Supplement: Supplement. — eMethods. Statistical Analysis eTable 1. Baseline Characteristics of Histology Group and Subset With Full Clinical Meta-Data eTable 2. Factors Associated With Resolution of NASH eTable 3. Baseline Characteristics by Fibrosis Evolution (Regressed, Stable, Progressed) eTable 4. Factors Associated With Progression to Advanced Fibrosis (Stage 3 or 4) in Patients With NAFLD on First Biopsy eTable 5. Factors Associated With Fibrosis Regression in Patients With Stage ≥1 Fibrosis and NAFLD on First Biopsy eTable 6. Histologic Features and Weight Change eFigure. Examples of Progression and Regression of Nonalcoholic Fatty Liver Disease [file jamanetwopen-2-e1912565-s001.pdf]

Supplementary Online Content

Kleiner DE, Brunt EM, Wilson LA, et al; Nonalcoholic Steatohepatitis Clinical Research Network. Association of histologic disease activity with progression of nonalcoholic fatty liver disease. *JAMA Netw Open*. 2019;2(10):e1912565. doi:10.1001/jamanetworkopen.2019.12565

eMethods. Statistical Analysis

eTable 1. Baseline Characteristics of Histology Group and Subset With Full Clinical Meta-Data

eTable 2. Factors Associated With Resolution of NASH

eTable 3. Baseline Characteristics by Fibrosis Evolution (Regressed, Stable, Progressed)

eTable 4. Factors Associated With Progression to Advanced Fibrosis (Stage 3 or 4) in Patients With NAFLD on First Biopsy

eTable 5. Factors Associated With Fibrosis Regression in Patients With Stage ≥1 Fibrosis and NAFLD on First Biopsy

eTable 6. Histologic Features and Weight Change

eFigure. Examples of Progression and Regression of Nonalcoholic Fatty Liver Disease

This supplementary material has been provided by the authors to give readers additional information about their work.

© 2019 Kleiner DE et al. *JAMA Network Open.*

eMethods: Statistical Analysis

We compared demographic, clinical, and laboratory data near the time of the initial biopsy by histologic diagnosis of patients at enrollment (NAFL, borderline, or definite steatohepatitis) using means (SD) or N (%) for the comparisons (Table 1). To determine the statistical significance of the comparisons, p-values were derived from analyses using Fisher's exact test for categorical measures or analysis of variance for continuous measures. Similar comparisons and analyses were performed to compare patients with fibrosis regression, no change, or fibrosis progression (eTable 3). Three separate multivariable linear regression analyses were performed to identify the clinical, laboratory, and histologic factors independently and parsimoniously related to change in fibrosis stage (Table 3). Each analysis included a different candidate set of factors, described below, and the best subset of factors fitting all possible regression models was determined based on the best the model with highest information index in relation to the change in fibrosis stage as the outcome in each model. The information index used was the Akaike Information Criteria (AIC), a penalized likelihood method that is a tradeoff between goodness of fit vs. model size, with smaller AICs corresponding to models with more information about the outcome[13, 14]. This approach is not based on p-values and avoids the need to adjust p-values for the multiplicity of candidate models. The candidate sets for each multiple linear regression model were defined as follows: 1.) Demographic, clinical, and histology (27 factors, 2^{27}=134,217,728 models): age at biopsy (years), sex, race, Hispanic ethnicity, diabetes status, metabolic syndrome; baseline and change from baseline BMI (kg/m^2), ALT (U/L), AST (U/L), alkaline phosphatase (U/L), LDL cholesterol (mg/dL), HDL cholesterol (mg/dL), triglycerides (mg/dL), and HOMA-IR (glucose [mmol/L] x insulin [µU/mL/22.5]); baseline steatosis grade, lobular inflammation grade, ballooning grade, portal inflammation grade, and fibrosis stage; 2.)

© 2019 Kleiner DE et al. *JAMA Network Open.*

Histologic features except NAS (9 factors, 2^9=512 models): baseline fibrosis stage, and baseline and change in steatosis grade, lobular inflammation grade, ballooning grade, portal inflammation grade, and; 3.) Histologic features including NAS (5 factors, 2^5=32 models): baseline fibrosis stage, and baseline and change in NAS and portal inflammation grade (the components of the NAS: steatosis, lobular inflammation, and ballooning were excluded). Several additional exploratory and sensitivity analyses were performed and shown in supplementary Tables 1-4. Supplementary Table 1 compares the demographic and histologic features of all patients with histologic data (N=446) to the subset of patients with clinical data within six months of the biopsy (N=219). In eTables 2, 4 and 5, clinical and histologic factors at baseline and changes over time (last vs. first biopsy) were compared for three alternative binary outcome measures of change in disease activity from first to last biopsy: 1.) resolution of NAFLD or NASH, 2.) progression to advanced fibrosis (stage 3 or 4), and 3.) fibrosis regression (any decrease in fibrosis stage in patients with stage ≥1 on first biopsy). P-values for baseline factors were derived from Fisher's exact test for categorical measures, and two-sample t-tests for continuous measures; p-values for changes over time were derived from ANCOVA models, adjusting for the baseline value of the clinical or histologic measure. Nominal (i.e., no adjustments for multiple comparisons), 2-sided p-values were considered significant if $P<0.05$. Analyses were performed using SAS software (version 9.4, SAS Institute, Cary, NC) and Stata (Release 15.1, Stata Corporation, College Station, TX).

© 2019 Kleiner DE et al. *JAMA Network Open.*

eTable 1: Baseline characteristics of histology group and subset with full clinical meta-data

Baseline Characteristics	All patients with histologic data (N=446)	Subset of patients with clinical data within 6 months of biopsy (N=219)	P
**Demographics**			
Age at biopsy – years	47 ± 12	47 ± 12	0.67
Male sex	152 (34%)	71 (32%)	0.67
White race	374 (88%)	182 (88%)	0.87
Hispanic or Latino	40 (9%)	22 (10%	0.65
Diabetes	149 (33%)	59 (27%)	0.09
**Histology**			
Steatosis	1.9 ± 0.8	2.0 ± 0.8	0.33
Lobular inflammation	1.6 ± 0.7	1.7 ± 0.7	0.66
Ballooning	1.1 ± 0.8	1.2 ± 0.8	0.53
Portal inflammation	1.1 ± 0.6	1.1 ± 0.6	0.84
Fibrosis stage	1.5 ± 1.2	1.4 ± 1.1	0.19
NAS*	4.7 ± 1.6	4.8 ± 1.6	0.30
Steatohepatitis diagnosis			0.75
No	86 (19%)	38 (17%)	
Borderline/indeterminate Zone 3 or Zone 1 pattern	84 (19%)	39 (18%)	
Yes, definite	276 (62%)	142 (65%)	
Interval between biopsies (years)	4.9 ± 2.8	3.9 ± 2.0	<0.001

* NAS=Nonalcoholic Fatty Liver Disease (NAFLD) Activity Score (sum of scores for steatosis, lobular inflammation, and ballooning, range 0-8)

© 2019 Kleiner DE et al. *JAMA Network Open.*

eTable 2. Factors associated with resolution of NASH

Factors associated with resolution of NASH	Resolution of NASH*			
	Yes	No	Mean difference (95% CI)	P†
**Baseline clinical factors**				
**N**	**40**	**141**		
ALT (U/L)	73 ± 46	87 ± 60	-14 (-35, 6)	0.10
AST (U/L)	48 ± 31	64 ± 50	-17 (-33, -0.3)	0.01
Alkaline Phosphatase (U/L)	84 ± 26	86 ± 24	-2 (-11, 7)	0.66
Insulin (μU/mL)‡	22.6 ± 12.6	28.3 ± 35.4	-5.7 (-17.1, 5.7)	0.12
Weight (kg)	103 ± 22	100 ± 20	3 (-4, 10)	0.41
BMI (kg/m^2)	36.1 ± 7.5	35.4 ± 6.3	0.7 (-1.7, 3.0)	0.58
Metabolic syndrome‡	33 (85%)	105 (75%)	--	0.28
Diabetes	10 (25%)	43 (31%)	--	0.56
Smoking history				
Current smoker	4 (10%)	15 (11%)	--	1.00
Ever smoked	18 (45%)	53 (38%)	--	0.46
Medication use				
Metformin	8 (20%)	35 (25%)	--	0.67
Vitamin E	5 (13%)	24 (17%)	--	0.63
Statins	7 (18%)	36 (26%)	--	0.40
**Baseline histology**				
**N**	**85**	**275**		
Steatosis	1.9 ± 0.8	2.0 ± 0.8	-0.1 (-0.3, 0.1)	0.59
Lobular inflammation	1.6 ± 0.7	1.8 ± 0.7	-0.3 (-0.4, -0.1)	0.003
Ballooning	1.3 ± 0.7	1.4 ± 0.7	-0.1 (-0.3, 0.0)	0.10
Mallory-Denk bodies	22 (26%)	119 (43%)	--	0.005
Portal inflammation	1.1 ± 0.6	1.2 ± 0.6	0.0 (-0.2, 0.1)	0.64
Fibrosis stage	1.4 ± 1.1	2.0 ± 1.1	-0.5 (-0.8, -0.3)	<0.001

© 2019 Kleiner DE et al. *JAMA Network Open.*

NAS	4.8 ± 1.4	5.2 ± 1.4	-0.5 (-0.8, -0.1)	0.009
Interval between biopsies (years)	5.0 ± 3.0	4.6 ± 2.7	0.4 (-0.2, 1.1)	0.20
**Change over time** (Δ= **last vs. first biopsy)**				
**Clinical factors**				
**N**	**40**	**141**		
Δ ALT (U/L)	-33 ± 47	-26 ± 59	-7 (-27, 13)	<0.001
Δ AST (U/L)	-20 ± 32	-14 ± 52	-6 (-23, 11)	<0.001
Δ Alkaline Phosphatase (U/L)	-5 ± 19	-5 ± 20	-1 (-7, 6)	0.74
Δ Insulin (μU/mL)	-4.1 ± 14.4	0.4 ± 37.9	-4.5 (-17.0, 8.1)	0.05
Δ Weight (kg)	-4.4 ± 10.1	1.2 ± 6.0	-5.6 (-8.1, -3.1)	<0.001
Δ BMI (kg/m^2)	-1.4 ± 3.6	0.5 ± 2.2	-1.8 (-2.7, -0.9)	<0.001
**Histology**				
**N**	**85**	**275**		
Δ Steatosis grade	-1.1 ± 1.0	-0.3 ± 0.9	-0.8 (-1.0, -0.5)	<0.001
Δ Lobular inflammation	-0.5 ± 0.8	-0.3 ± 0.9	-0.2 (-0.4, 0.0)	<0.001
Δ Ballooning	-1.2 ± 0.8	-0.1 ± 0.9	-1.2 (-1.4, -0.9)	<0.001
Mallory-Denk bodies				<0.001
Improved	21 (25%)	47 (17%)		
Worsened	1 (1%)	47 (17%)		
No change	63 (74%)	181 (66%)		
Δ Portal inflammation	0.0 ± 0.7	0.1 ± 0.6	-0.2 (-0.3, 0.0)	0.008
Δ Fibrosis	-0.8 ± 1.1	0.3 ± 1.1	-1.0 (-1.3, -0.7)	<0.001
Δ NAS	-2.8 ± 1.7	-0.7 ± 1.8	-2.2 (-2.6, -1.7)	<0.001

* Resolution of NASH defined as a diagnosis of NAFLD only or not NAFLD at second biopsy among patients with borderline/definite steatohepatitis on first biopsy. Note that the N for the clinical and laboratory data is smaller because only those data collected within 6 months of the biopsy were included. All participants in the cohort are included for the data on histology.

© 2019 Kleiner DE et al. *JAMA Network Open.*

†P-values for baseline factors derived from two-sample t-tests for continuous measures and Fisher's exact test for categorical measures. P-values for changes over time derived from ANCOVA models, adjusting for the baseline value of the clinical or histologic measure.
‡ N=2 participants missing insulin and HOMA-IR
ALT=Alanine Aminotransferase
AST=Aspartate Aminotransferase
BMI=Body Mass Index
NAS=Nonalcoholic Fatty Liver Disease (NAFLD) Activity Score (sum of scores for steatosis, lobular inflammation, and ballooning, range 0-8)

© 2019 Kleiner DE et al. *JAMA Network Open.*

eTable 3. Baseline characteristics by fibrosis evolution (regressed, stable, progressed)

Baseline characteristics	Fibrosis regressed	Fibrosis Stable	Fibrosis progressed	P*
**Demographics**				
**N**	**132**	**163**	**151**	
Age at biopsy – years	47 ± 11	47 ± 12	48 ± 10	0.86
Male Sex	46 (35%)	57 (35%)	49 (32%)	0.88
White race†	102 (82%)	138 (90%)	134 (91%)	0.04
Hispanic or Latino	14 (11%)	18 (11%)	8 (5%)	0.13
Diabetes	48 (36%)	54 (33%)	47 (31%)	0.66
Smoking history				
Current smoker	5 (4%)	17 (10%)	18 (12%)	0.03
Ever smoked	51 (39%)	55 (34%)	57 (38%)	0.63
**Clinical and laboratory data (collected within 6 months of biopsy)**				
**N**	**64**	**84**	**71**	
Metabolic syndrome†	45 (70%)	64 (76%)	50 (72%)	0.72
BMI (kg/m^2)	35.1 ± 6.3	35.6 ± 7.4	35.2 ± 6.2	0.88
ALT (U/L)	83 ± 66	78 ± 48	75 ± 52	0.69
AST (U/L)	54 ± 41	56 ± 37	60 ± 54	0.75
Alkaline Phosphatase (U/L)	85 ± 23	84 ± 27	89 ± 20	0.74
LDL-C (mg/dL)	120 ± 34	124 ± 37	125 ± 37	0.70
HDL-C (mg/dL)	42 ± 10	42 ± 11	45 ± 10	0.21
Triglycerides (mg/dL)	179 ± 117	177 ± 86	187 ± 122	0.85
Glucose (mg/dL)	102 ± 26	104 ± 31	103 ± 36	0.91
Insulin (µU/mL)†	20 ± 11	27 ± 32	28 ± 37	0.25
HOMA-IR (glucose [mmol/L] x insulin [µU/mL/22.5)†	5.4 ± 4.5	7.8 ± 12.0	7.3 ± 9.8	0.31
Medication use				
Metformin	17 (27%)	19 (23%)	10 (14%)	0.17
Vitamin E	7 (11%)	18 (21%)	8 (11%)	0.14
Statins	16 (25%)	18 (21%)	16 (23%)	0.90
**Histology**				
**N**	**132**	**163**	**151**	
Steatosis	2.0 ± 0.8	1.8 ± 0.8	1.9 ± 0.9	0.02
Lobular inflammation	1.8 ± 0.7	1.6 ± 0.7	1.6 ± 0.7	0.08
Ballooning	1.3 ± 0.8	1.1 ± 0.9	1.1 ± 0.8	0.07
Mallory-Denk bodies	42 (32%)	51 (31%)	48 (32%)	1.00
Portal inflammation	1.1 ± 0.6	1.1 ± 0.6	1.0 ± 0.6	0.43
Fibrosis stage	2.0 ± 0.9	1.5 ± 1.4	1.2 ± 1.0	<0.001
NAS	5.1 ± 1.5	4.4 ± 1.7	4.6 ± 1.5	0.002
Interval between biopsies - years	4.6 ± 2.6	4.7 ± 3.0	5.4 ± 2.8	0.02
Biopsy length (mm)	19.6 ± 9.5	18.3 ± 8.6	18.0 ± 9.6	0.30

*P-values derived from Fisher's exact test for categorical variables and ANOVA for continuous measures. Note that the N for the clinical and laboratory data is smaller because only those data collected within 6 months of the biopsy were included. All participants in the cohort are included for the data on histology.
† N=21 participants refused to state race; N=3 participants missing metabolic syndrome, insulin, and HOMA-IR; N=4 participants missing LDL cholesterol.

© 2019 Kleiner DE et al. *JAMA Network Open.*

BMI=Body Mass Index
ALT=Alanine Aminotransferase
AST=Aspartate Aminotransferase
LDL-C=Low Density Lipoprotein Cholesterol
HDL-C=High Density Lipoprotein Cholesterol
HOMA-IR=Homeostatic Model Assessment of Insulin Resistance
NAS=Nonalcoholic Fatty Liver Disease (NAFLD) Activity Score (sum of scores for steatosis, lobular inflammation, and ballooning, range 0-8)

© 2019 Kleiner DE et al. *JAMA Network Open.*

eTable 4. Factors associated with progression to advanced fibrosis (stage 3 or 4) in patients with NAFLD on first biopsy

**Factors associated with progression to advanced fibrosis**	**Progression to advanced fibrosis (stage 3 or 4)**			
	**Yes**	**No**	**Mean difference (95% CI)**	**P***
**Baseline clinical factors**				
**N**	**21**	**152**		
ALT (U/L)	91 ± 45	70 ± 46	21 (0, 42)	0.05
AST (U/L)	75 ± 57	48 ± 39	26 (8, 45)	0.05
Alkaline Phosphatase (U/L)	88 ± 20	83 ± 23	5 (-5, 15)	0.35
Insulin (µU/mL)†	23.9 ± 15.0	23.1 ± 25.7	0.8 (-10.9, 12.4)	0.85
Weight (kg)	91 ± 15	99 ± 21	-8 (-17, 2)	0.11
BMI (kg/m^2)	34.5 ± 6.1	35.1 ± 6.7	-0.5 (-3.6, 2.5)	0.73
Metabolic syndrome†	20 (95%)	108 (72%)	--	0.03
Diabetes	7 (33%)	35 (23%)	--	0.29
Smoking history				
Current smoker	3 (14%)	12 (8%)	--	0.40
Ever smoked	11 (52%)	52 (34%)	--	0.15
Medication use				
Metformin	4 (19%)	26 (17%)	--	0.76
Vitamin E	3 (14%)	22 (14%)	--	1.00
Statins	6 (29%)	34 (22%)	--	0.58
**Baseline histology**				
**N**	**54**	**268**		
Steatosis	1.9 ± 0.9	2.0 ± 0.8	0.0 (-0.3, 0.2)	0.81
Lobular inflammation	1.7 ± 0.6	1.5 ± 0.7	0.2 (0.0, 0.4)	0.07
Ballooning	1.4 ± 0.8	0.8 ± 0.8	0.5 (0.3, 0.8)	<0.001
Mallory-Denk bodies	24 (44%)	36 (13%)	--	<0.001
Portal inflammation	1.1 ± 0.5	0.9 ± 0.6	0.3 (0.1, 0.4)	0.001

© 2019 Kleiner DE et al. *JAMA Network Open.*

Fibrosis stage	1.4 ± 0.7	0.8 ± 0.8	0.5 (0.3, 0.8)	<0.001
NAS	5.0 ± 1.4	4.3 ± 1.6	0.7 (0.2, 1.1)	0.005
Interval between biopsies (years)	5.7 ± 2.7	4.9 ± 2.8	0.8 (0.0, 1.7)	0.04
**Change over time (Δ= last vs. first biopsy)**				
**Clinical factors**				
**N**	**21**	**152**		
Δ ALT	-14 ± 48	-20 ± 46	6 (-15, 27)	<0.001
Δ AST	-6 ± 60	-11 ± 41	5 (-15, 25)	<0.001
Δ Alkaline Phosphatase	-6 ± 24	-6 ± 16	0 (-8, 7)	0.73
Δ Insulin	5.8 ± 19.9	-1.5 ± 30.6	7 (-7, 21)	0.06
Δ Weight (kg)	-0.1 ± 5.8	0.1 ± 7.8	-0.2 (-3.7, 3.3)	0.83
Δ BMI (kg/m^2)	-0.1 ± 2.4	0.1 ± 2.8	-0.2 (-1.5, 1.1)	0.75
**Histology**				
**N**	**54**	**268**		
Δ Steatosis grade	-0.4 ± 0.9	-0.5 ± 1.0	0.1 (-0.2, 0.3)	0.74
Δ Lobular inflammation	0.0 ± 0.9	-0.3 ± 0.8	0.3 (0.0, 0.5)	<0.001
Δ Ballooning	0.3 ± 1.1	-0.2 ± 1.0	0.4 (0.1, 0.7)	<0.001
Mallory-Denk bodies				<0.001
Improved	10 (19%)	22 (8%)	--	
Worsened	19 (35%)	21 (8%)	--	
No change	25 (46%)	225 (84%)	--	
Δ Portal inflammation	0.4 ± 0.8	0.1 ± 0.7	0.3 (0.1, 0.5)	<0.001
Δ NAS	-0.2 ± 2.0	-0.9 ± 2.0	0.7 (0.2, 1.3)	<0.001

* P-values for baseline factors derived from two-sample t-tests for continuous measures and Fisher's exact test for categorical measures. P-values for changes over time derived from ANCOVA models, adjusting for the baseline value of the clinical or histologic measure. Note that the N for the clinical and laboratory data is smaller because only those data collected within 6 months of the biopsy were included. All participants in the cohort are included for the data on histology.
† N=2 participants missing insulin and metabolic syndrome.
ALT=Alanine Aminotransferase

© 2019 Kleiner DE et al. *JAMA Network Open.*

AST=Aspartate Aminotransferase
BMI=Body Mass Index
NAS=Nonalcoholic Fatty Liver Disease (NAFLD) Activity Score (sum of scores for steatosis, lobular inflammation, and ballooning)

© 2019 Kleiner DE et al. *JAMA Network Open.*

eTable 5. Factors associated with fibrosis regression* in patients with stage ≥1 fibrosis and NAFLD on first biopsy

Factors associated with fibrosis regression	Fibrosis Regression			
	Yes	No	Mean difference (95% CI)	P†
**Baseline clinical factors**				
**N**	**64**	**100**		
ALT (U/L)	83 ± 66	88 ± 55	-5 (-23, 14)	0.62
AST (U/L)	54 ± 41	69 ± 52	-15 (-30, 0)	0.05
Alkaline Phosphatase (U/L)	85 ± 23	89 ± 25	-4 (-11, 4)	0.34
Insulin (µU/mL)‡	20.1 ± 10.9	32.7 ± 40.8	-12.5 (-22.9, -2.2)	0.02
Weight (kg)	99 ± 20	100 ± 20	-0.6 (-7.0, 5.7))	0.84
BMI (kg/m^2)	35.0 ± 6.3	35.7 ± 6.8	-0.7 (-2.7, 1.4)	0.53
Metabolic syndrome‡	45 (70%)	79 (80%)	--	0.19
Diabetes	21 (33%)	29 (29%)	--	0.61
Smoking history				
Current smoker	2 (3%)	12 (12%)	--	0.08
Ever smoked	23 (36%)	37 (37%)	--	1.00
Medication use				
Metformin	17 (27%)	22 (22%)	--	0.57
Vitamin E	7 (11%)	22 (22%)	--	0.09
Statins	16 (25%)	23 (23%)	--	0.85
**Baseline Histology**				
**N**	**132**	**206**		
Steatosis	2.0 ± 0.8	1.8 ± 0.9	0.2 (0.0, 0.4)	0.02
Lobular inflammation	1.8 ± 0.7	1.8 ± 0.7	0.0 (-0.2, 0.1)	0.83
Ballooning	1.3 ± 0.8	1.5 ± 0.7	-0.2 (-0.3, 0.0)	0.05
Mallory-Denk bodies	42 (32%)	99 (48%)	--	0.003
Portal inflammation	1.1 ± 0.6	1.2 ± 0.6	-0.1 (-0.2, 0.0)	0.19
Fibrosis stage	2.0 ± 0.9	2.1 ± 1.0	-0.1 (-0.3, 0.1)	0.32

© 2019 Kleiner DE et al. *JAMA Network Open.*

NAS	5.1 ± 1.5	5.1 ± 1.5	0.0 (-0.3, 0.3)	0.91
Interval between biopsies (years)	4.6 ± 2.6	4.8 ± 3.0	-0.2 (-0.8, 0.4)	0.53
**Change over time** (Δ= **last vs. first biopsy)**				
**Clinical factors**				
**N**	**64**	**100**		
Δ ALT	-39 ± 63	-23 ± 53	-16 (-34, 3)	<0.001
Δ AST	-21 ± 39	-14 ± 53	-7 (-22, 9)	0.001
Δ Alkaline Phosphatase	-9 ± 18	-5 ± 22	-4 (-10, 2)	0.08
Δ Insulin	0.5 ± 18.4	-1.1 ± 43.1	2 (-10, 13)	0.08
Δ Weight	-0.6 ± 7.2	0.4 ± 6.0	-1.1 (-3.1, 1.0)	0.31
Δ BMI (kg/m^2)	-0.2 ± 2.4	0.2 ± 2.2	-0.5 (-1.2, 0.2)	0.19
**Histology**				
**N**	**132**	**206**		
Δ Steatosis grade	-0.8 ± 1.0	-0.3 ± 0.9	-0.5 (-0.7, -0.3)	<0.001
Δ Lobular inflammation	-0.5 ± 0.8	-0.2 ± 0.9	-0.3 (-0.5, -0.1)	<0.001
Δ Ballooning	-0.7 ± 1.1	-0.1 ± 0.9	-0.6 (-0.8, -0.4)	<0.001
Mallory-Denk bodies				0.004
Improved	31 (23%)	37 (18%)		
Worsened	8 (6%)	37 (18%)		
No change	93 (70%)	132 (64%)		
Δ Portal inflammation	-0.1 ± 0.7	0.2 ± 0.6	-0.3 (-0.4, -0.1)	<0.001
Δ NAS	-2.0 ± 2.1	-0.6 ± 1.9	-1.4 (-1.8, -1.0)	<0.001

* Fibrosis regression among participants with stage ≥1 fibrosis and NAFLD on first biopsy defined as a one stage or more decrease in fibrosis stage at last biopsy, with a change from 1b to 1a fibrosis considered fibrosis regression.

† P-values for baseline factors derived from two-sample t-tests for continuous measures and Fisher's exact test for categorical measures. P-values for changes over time derived from ANCOVA models, adjusting for the baseline value of the clinical or histologic measure

‡ N=2 participants missing insulin, 1 participant missing metabolic syndrome.

Note that the N for the clinical and laboratory data is smaller because only those data collected within 6 months of the biopsy were included. All participants in the cohort are included for the data on histology.

© 2019 Kleiner DE et al. *JAMA Network Open.*

ALT=Alanine Aminotransferase
AST=Aspartate Aminotransferase
BMI=Body Mass Index
NAS=Nonalcoholic Fatty Liver Disease (NAFLD) Activity Score (sum of scores for steatosis, lobular inflammation, and ballooning, range 0-8)

© 2019 Kleiner DE et al. *JAMA Network Open.*

eTable 6. Histologic features and weight change

	Weight change (kg)			
Histologic Feature	Weight loss of >5kg (N=39)	5kg loss – 5 kg gain (N=129)	Weight gain of >5kg (N=50)	P*
**Change over time (Δ=last vs. first biopsy)**				
Δ Steatosis grade	-0.8 ‡ 1.0	-0.3 ‡ 0.9	-0.1 ‡ 0.8	0.001
Δ Lobular inflammation	-0.6 ‡ 0.9	-0.3 ‡ 0.7	-0.2 ‡ 0.8	0.02
Δ Ballooning	-0.7 ‡ 0.9	-0.2 ‡ 0.9	+0.1 ‡ 1.1	<0.001
Δ Portal inflammation	0.0 ‡ 0.8	0.1 ‡ 0.6	0.1 ‡ 0.6	0.18
Δ Fibrosis stage	-0.3 ‡ 1.0	+0.1 ‡ 1.0	+0.1 ‡ 1.1	0.06
Δ NAS	-2.0 ‡ 1.8	-0.8 ‡ 1.7	-0.2 ‡ 1.9	<0.001

* P for trend. Analysis limited to subset of 218 participants with non-missing weight measurements within 6 months of biopsy (one participant was missing weight measurement at second biopsy).

NAS=Nonalcoholic Fatty Liver Disease (NAFLD) Activity Score (sum of scores for steatosis, lobular inflammation, and ballooning, range 0-8)

© 2019 Kleiner DE et al. *JAMA Network Open.*

eFigure. Examples of Progression and Regression of Nonalcoholic Fatty Liver Disease

A. Progression

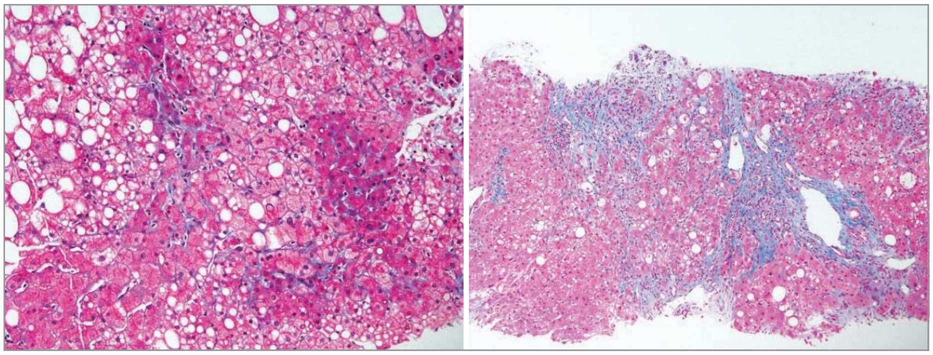

B. Regression

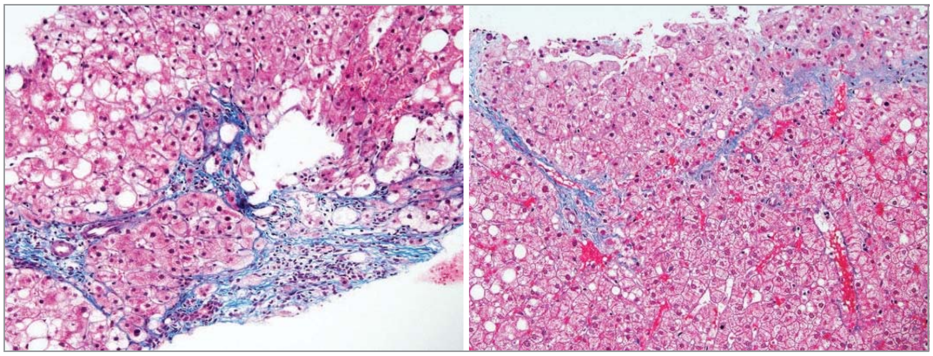

Masson trichrome stains demonstrating either progression (A) or regression (B) of fibrosis. Progression of NAFLD (from left to right panel) occurred over 7.75 years from initial biopsy with mild activity (NAFLD activity score [NAS], 3) and early stage 2 fibrosis (only perisinusoidal fibrosis shown), progressing to the follow-up biopsy that had marked activity (NAS, 7) and extensive perisinusoidal fibrosis with bridging fibrosis (stage 3). The NAS is the sum of scores for steatosis, lobular inflammation, and ballooning, range 0 to 8, with 8 indicating more severe disease. Regression of NAFLD (from left to right panel) occurred over 3.13 years from an initial biopsy with moderate activity (NAS, 5) and bridging fibrosis (stage 3) to a follow-up biopsy with mild activity (NAS, 2) and only perisinusoidal fibrosis (stage 1). (Masson trichrome, original magnification, A: left, ×200, right, ×100 and B: left, ×200, right, ×200).

© 2019 Kleiner DE et al. *JAMA Network Open.*
